# Targetoid‐like lesions and chilblain‐like erythema manifested on hands and feet: A case of Rowell syndrome from China

**DOI:** 10.1002/iid3.979

**Published:** 2023-08-28

**Authors:** Yanqiao Li, Shi Cheng, Chuanpeng Ying, Ling Li, Guangxing Wang, Xuejun Chen

**Affiliations:** ^1^ Department of Dermatology, Sichuan Provincial People's Hospital, School of Medicine University of Electronic Science and Technology of China Chengdu China; ^2^ School of Medicine Tongji University Shanghai China

**Keywords:** erythema multiforme, lupus erythematosus, Rowell syndrome

## Abstract

**Background**: Rowell syndrome (RS) is an uncommon condition characterized by erythema multiforme (EM)‐like lesions and lupus erythematosus. It is more common in females, and EM may be the first manifestation of the disease with positive autoantibodies, such as antinuclear antibody (ANA), SSA, SSB and rheumatoid factor. The pathogenesis of RS is unknown and is likely caused by drug induction, ultraviolet exposure and infection. **Method**: We describe a case of RS from China which presented as characteristic targetoid‐like lesions and chilblain‐like erythema on hands and feet. This is a case of RS in a female patient from the inpatient department of dermatology. **Results**: A 41‐year‐old female with systemic lupus erythematosus exhibited chilblain‐like erythema and characteristic EM lesions on her extremities. She tested positive for serum ANA (1:320) and anti‐double‐stranded DNA, as well as other autoantibodies. Systemic glucocorticoids and hydroxychloroquine worked effectively for her. **Conclusion**: The present case met diagnostic criteria of RS. Notably, there was a co‑occurrence of facial butterfly erythema, chilblain‐like erythema and EM lesions distributed on the limbs in this case.

## Graphical abstract

The Rowell syndrome was described by the authors in a 41‐year‐old female from China. Targetoid‐like lesions and chilblain‐like erythema manifested on her hands and feet were characteristic and she responded favorably to systemic glucocorticoid and hydroxychloroquine.

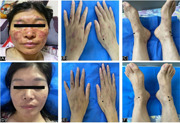

## 1 INTRODUCTION

Rowell Syndrome (RS) is a syndrome characterized by rashes of lupus erythematosus (LE) and suggestive erythema multiforme (EM)‐like cutaneous lesions along with specific immunological abnormalities.^1^ The disease was first reported by Rowell^1^ in 1963, redefined the diagnostic criteria by Zeitouni et al.^2^ in 2000 and considered to be a possible special type of lupus erythematosus. Patients with RS often present with discoid lupus erythematosus (DLE), subacute cutaneous lupus erythematosus (SCLE), or systemic lupus erythematosus (SLE) before the onset of EM lesions, with intervals ranging from 6 weeks to 14 years.^3^ It has been disputed as to whether RS is a unique disease or just a coexistence of LE and EM concurrently as a subset of LE. The precise etiopathogenesis of RS remains unclear; however, it is believed that it may be triggered by drug, infection, ultraviolet exposure, cigarette smoking, and psychological stress.^4^ Corticosteroids, hydroxychloroquine, immunosuppression, and biologic treatments (such as anifrolumab) are among the therapeutic options for RS.^5^ SLE is a chronic autoimmune‐mediated inflammatory disorder with multisystem and multiorgan involvement. Frostbite‐like lesions in the extremities are frequently significant clinical symptoms of vasculitis in SLE, but the limbs do not typically develop multiform erythematous skin lesions. EM is an acute, immune‐mediated condition linked to infection, medications and autoimmune disorder without special autoantibody. It is distinguished by evident target lesions on the skin, which are usually accompanied by erosion, blisters, or bullae of mucosal areas (such as the mouth, genitals, and eyes). In clinical practice, the overlap of SLE with EM is uncommon and rarely reported among Chinese people in the existing knowledgebase. Herein, we present a case of RS from a Chinese female patient with SLE who exhibited chilblain‐like erythema^6^ and characteristic EM lesions^7^ on her hands and feet. In addition to the indicative skin lesions, she exhibited multiple immunological abnormalities, including positive antinuclear antibody (ANA) (speck type), anti‐dsDNA antibodies, and lupus transaminitis, and responded well to glucocorticoids and hydroxychloroquine without an administration of immunosuppressive drugs or biological agents. The presentation, in this case, might support the contention additionally that RS is a subset of LE rather than an independent illness.

## 2 CASE

**Figure 1:**
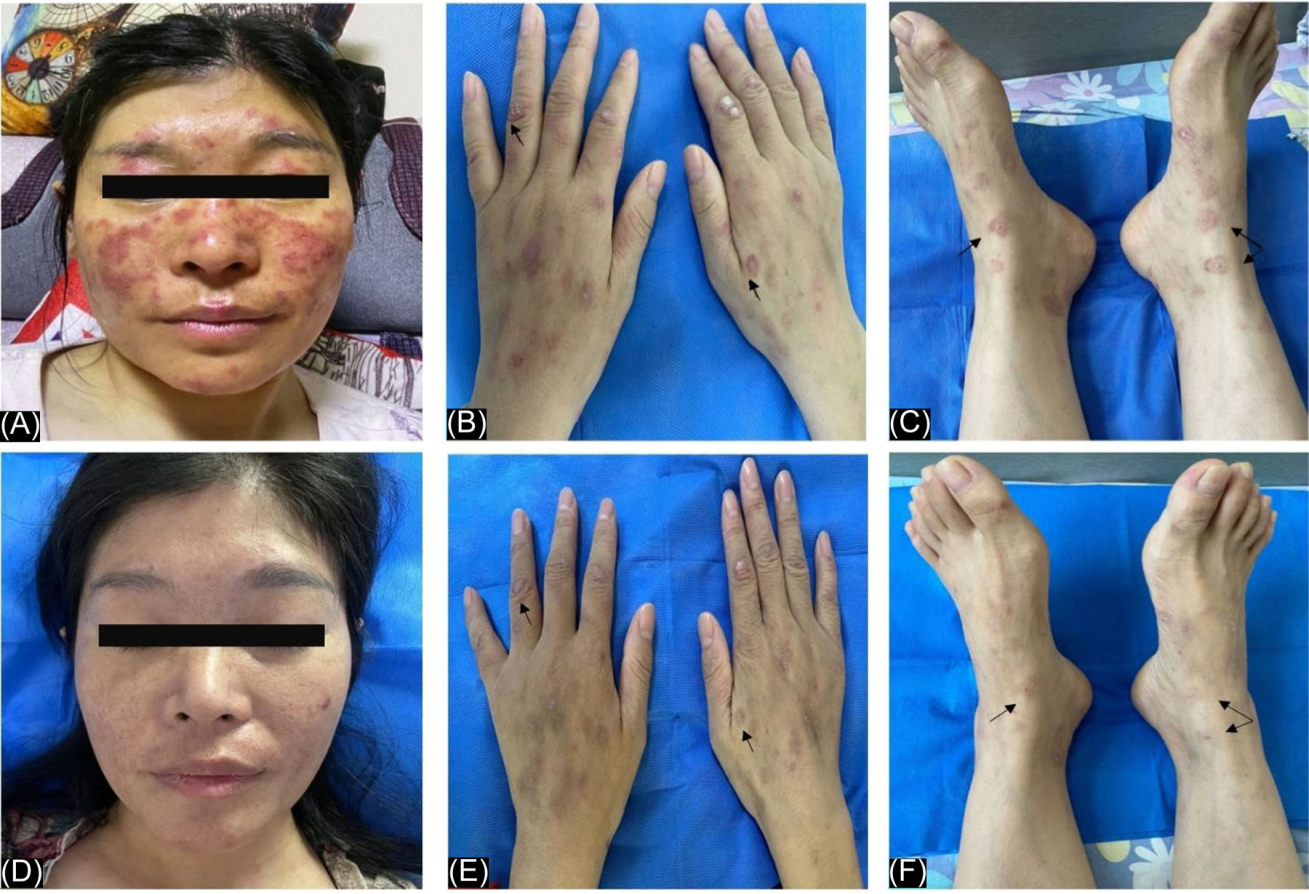
Features of lesions of Rowell's syndrome in the case before and after treatment. A 41‐year‐old woman presented with classic facial butterfly erythema (A), chilblain‐like erythema on both hands which was marked with a black arrow (B), and targetoid‐like lesions of EM indicated by the black arrows on both feet (C). After receiving therapy for 2 weeks, these skin lesions significantly improved (D, E, F).

**Figure 2:**
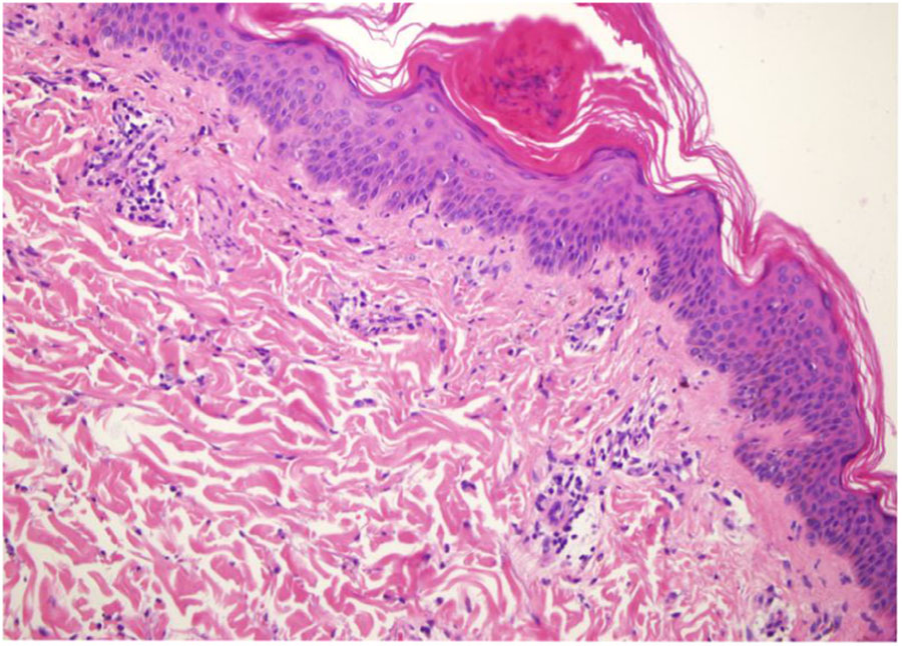
Histopathology of the skin lesions showed liquefaction degeneration of basal cells, pigment incontinence, and infiltration of perivascular inflammatory cells in the superficial dermis (HE, ×200).

A 41‐year‐old Chinese woman presented with facial butterfly erythema for one year (Figure 1A). Half a year ago, she developed chilblain‐like erythema which manifested as purplish red papules, nodules, or plaques (Figure 1B) and skin lesions of EM with target shaped manifestation (Figure 1C) on the extended side of the skin of her hands and feet, respectively. In addition, she suffered from discoid erythema on the auricle, alopecia, oral ulcer, and photosensitivity. She had no family history of SLE and other comorbidity such as high blood pressure, coronary heart disease, diabetes, and so forth. She was initially diagnosed with EM in an out‐patient hospital, and the skin lesions did not improve after conventional antiallergic therapy. After an admission, the patient was tested for novel coronavirus (SARS‐Cov‐2) nucleic acid test to exclude the COVID‐19‐associated dermatologic manifestations^8^ and the result was negative. Enlarged bilateral cervical and axillary lymph nodes were detected on ultrasound. Leucopenia (white blood cell count, 1.93 × 10^9^/L), neutropenia (neutrophil count, 1.56 × 10^9^/L), anemia (hemoglobin, 113 g/L), and thrombopenia (96 × 10^9^/L) were found in blood routine. The erythrocyte sedimentation rate (51 mm/h) was increased, and C‑reactive protein (<0.5 mg/L) was normal. The serum activity of aminotransferases (alanine aminotransferase, 94 U/L and aspartate aminotransferase, 136 U/L) and creatine kinase (lactate dehydrogenase, 310 U/L) was increased. she was tested for serum ANA (1:320, speckled pattern) and positive for anti‐double‐stranded DNA, anti‐nucleosome antibody, anti‐histone antibody, and antimitochondrial antibody‐M2 antibodies. Both C3 and C4 complement fractions significantly decreased (C3, 0.34 g/L; C4, <0.07 g/L). Tests for circulating anti *Ro*/*La* antibodies and *RF* were negative. Routine urine examination revealed that she had proteinuria (1+), urinary epithelial cells (1+), and white blood cells were mildly elevated (28/μL). The 24‐h urinary protein quantification was 0.54 g. Biopsy from erythema of the upper extremity showed liquefaction degeneration of basal cells, pigment incontinence, and infiltration of perivascular inflammatory cells in the superficial dermis, consistent with LE (Figure 2). Direct immunofluorescence of skin lesions showed positive deposition of IgG along the basement membrane. She did not present with fever and her chest CT showed no significant abnormalities. The patient generally exhibited the following clinical manifestations: (1) skin and mucous membrane involvement (facial butterfly erythema, chilblain‐like erythema, EM, alopecia, oral ulcer, and photosensitivity) (2) blood system involvement (leukopenia, anemia, thrombocytopenia) (3) proteinuria (>0.5 g/24 h) (4) positive immunological indicators (positive ANA, positive anti‐dsDNA antibodies, and hypocomplementemia) (5) proteinuria and lupus transaminitis. We found no evidence of infection or malignancy, and she was diagnosed with SLE. Furthermore, according to the diagnostic criteria of Rowell and Zeitouni et al.,^1^, ^9^ this patient met three major criteria (SLE, EM‐like lesions, and speckled ANA pattern) and one minor criterion (chilblain‐like erythema), and she was eventually diagnosed with RS. In addition, excluding viral infection, fatty liver, and drug‐induced liver insufficiency, this case was also recognized as lupus transaminitis, which was consistent with a previous case report.^10^ After receiving a dosage of intravenous methylprednisolone (equivalent to 1 mg/kg/day of prednisolone) for 2 weeks followed by oral prednisolone (40 mg/day), oral hydroxychloroquine 200 mg twice daily, and topical desonide cream, the patient's rash subsided significantly (Figures 1D−F) with no fresh lesions, and the related re‐examination indicators improved gradually, including the disappearance of proteinuria. The patient's indicators remained stable without a rebound when the glucocorticoid was reduced to 40 mg/day prednisone. She is currently under treatment and follow‐up.

## 3 DISCUSSION

In clinic, EM complicates lupus erythematosus quite seldom. In 1963, Rowell et al.^1^ summarized the clinical and immunological features of patients with lupus erythematosus complicated by EM lesions and named RS. The diagnostic standards for RS were modified by Zeitouni et al.^9^ including three main symptoms: Lupus (systemic, subacute or discoid lupus erythematosus), erythematous pleomorphic lesion (involving or not involving mucosa), and positive ANA, spotted type. Minor symptoms were frostbite‐like lesions, positive anti‐*Ro* (SSA) or anti‐*La* (SSB) antibodies, and positive rheumatoid factor (RF). RS can be diagnosed by three main symptoms and one minor symptom. The clinical feature of this case was that the EM on her limbs occurred on basis of the lesion of SLE for half a year. According to this diagnostic criterion, the diagnosis of RS was conformed, which indicated that this was a case of LE complicated with RS.

In recent years, it is controversial whether RS is an independent disease. Several investigators believe RS is a subtype of subacute lupus erythematosus, and clinical and histological distinctions between them are challenging. Histopathology alterations were not included in Zeitouni et al's diagnostic criteria. Histopathological changes of RS are not consistent. The pathological evaluation of the skin lesion might reveal EM^11^ and/or lupus erythematosus‐like manifestations^12^; nevertheless, the major pathological feature of our case is LE lesion. Overall, in addition to being diagnosed with SLE, our case fully meets the diagnostic criteria for RS. We believe that RS is more likely to be a component of LE rather than an individual disorder in light of the histological findings of the skin lesions and autoimmune antibody results. We compare the characteristics of this case with the pertinent literature cases previously reported in the past decade, as shown in Table 1. Generally speaking, among these reported cases, 28% of the patients were from India, 64% of them were female, and 60% exhibited SLE. The two most frequently used medications were glucocorticoids and hydroxychloroquine.

**Table 1:** Clinical and immunological characteristics of this patient and Rowell's syndrome reported in literature in the past decade.

	Gender	Age (year)	Type	EM	MI	ANA	PL	Anti‐ Ro/Lo	RF	Treatment	Patient's country	Reference
[^a^]	F	41	SLE	+	−	+, Sp 1:320	+	−	−	methylprednisolone (1 mg/kg/day), prednisolone (40 mg/day), HCQ (400 mg/day)	China	ND
01	M	10	SLE	+	lips, oral, pharyngeal mucosa	+, Sp 1:1280	+	Ro + /Lo+	+	methyl‐prednisolone (30 mg/kg/day), prednisolone (2 mg/kg/day)	India	Rheumatol Int. 2013;33(4):1075‐7.
02	F	70	SCLE	+	/	+, Sp 1:1280	/	Ro + /Lo+	+	prednisone (25 mg/day), HCQ (400 mg/day)	Italy	J Dtsch Dermatol Ges. 2014;12(11):1039‐42.
03	M	15	SLE	+	lips, oral mucosa	+, Sp /	+	Ro + /Lo+	−	prednisolone (2 mg/kg/day), HCQ, AZA	India	Indian Dermatol Online J. 2014;5(Suppl1):S33‐5.
04	M	33	DLE	+	−	+, Sp ＞20	−	−	/	prednisolone(40 mg), HCQ (400 mg/day), AZA (50 mg)	India	Indian Dermatol Online J. 2015;6(Suppl1):S12‐6.
05	F	42	DLE	+	−	+, Sp 1:320	/	−	+	prednisolone (1 mg/kg/day), HCQ (400 mg/day)	Italy	GITAL DERMATOLVENEREOL 2015;150:1‐2.
06	F	37	SCLE	/	Lips, eyes	+, /	/	Ro + /Lo+	−	intravenous corticosteroids, HCQ (400 mg/day), AZA (100 mg/day)	Columbia	Cutis. 2017;100(1):E8‐E11.
07	M	22	SLE	+	hard palate, buccal mucosa	+, Sp 1:160	−	Ro+	+	prednisolone (50 mg/day), HCQ (400 mg/day), pulse CTX (700 mg)	India	J Assoc Physicians India. 2018;66(1):98‐9.
08	F	67	SCLE	+	−	+, Sp 1:1280	/	Ro + /Lo+	/	methylprednisolone (8 mg/day), HCQ (400 mg/day),	Bulgaria	Acta Dermatovenerol Croat. 2019;27(2):124‐126.
09	M	61	SLE	+	/	+, Sp	/	Ro + /Lo+	/	prednisolone (40 mg/day), HCQ (400 mg/day), MMF (1 g/day)	Spain	SAGE Open Med Case Rep. 2019;7:2050313×19847337.
10	F	26	SLE	+	oral mucosa	+, Sp 1:320	/	/	/	high‐dose steroids, HCQ (400 mg/day), MMF (2 g/day)	Nigeria	Acta Dermatovenerol Croat. 2019;27(3):200‐201.
11	M	11	SLE	+	oral mucosa	+, /	/	Ro + /Lo+	/	methylprednisolone, prednisolone, HCQ, AZA	PAK	Cureus. 2019;11(5):e4604.
12	F	30	SLE	+	oral mucosa	+, /	/	Ro + /Lo+	−	prednisolone (40 mg/day), HCQ (400 mg/day), AZA (150 mg/day)	Saudi Arabia	Open Access Rheumatol. 2020;12:91‐96.
13	M	17	SLE	+	−	+, /	/	Ro+	/	prednisolone(1 mg/kg/day), HCQ (400 mg/day)	Italy	J Clin Aesthet Dermatol. 2020;13(4):40‐42.
14	M	20	LE	+	−	+, Sp 1:1280		Ro + /Lo+	+	prednisolone	/	Case Rep Rheumatol. 2020;2020:8884230.
15	F	18	/	+	oral mucosa	+, /	/	Ro+	+	prednisolone (1 mg/kg/day), HCQ (200 mg/day)	India	BMJ Case Rep. 2020;13(9):e235173.
16	F	39	SLE	+	−	/	/	Ro+	−	/	USA	The Journal of Rheumatology2020;47:2.
17	F	66	SLE	+	−	+, Hm 1:360	/	−	/	prednisolone (60 mg/day), HCQ (400 mg/day), MMF, belimumab	USA	Dermatol Online J. 2021;27(2):13030.
18	M	15	SCLE	+	oral mucosa	+, Sp	/	Ro+	‐	dexamethasone (8 mg), HCQ (400 mg/day)	India	J Postgrad Med. 2021;67(2):109‐112.
19	F	26	SLE	+	lips	+, Sp	+	Ro+	−	prednisolone (1 mg/kg/day), rituximab (375 mg/m^2^)	USA	Case Rep Rheumatol. 2021;2021:2727382.
20	F	43	SLE	/	−	/	/	/	/	prednisolone (40 mg/day), HCQ (400 mg/day), IVIG MMF (1 g/day), rituximab	USA	JAAD Case Rep. 2022;31:27‐30.
21	F	32	SLE	/	−	+, Sp	/	/	/	methylprednisolone (125 mg), prednisolone (40 mg/day), HCQ (200 mg/day)	USA	Mod Rheumatol Case Rep. 2022;6(1):33‐35.
22	F	67	SCLE	+	−	−	/	Ro+	−	methylprednisolone (40 mg/day), HCQ (400 mg/day)	Caucasian	Front Med (Lausanne). 2022;9:815743.
23	M	10	LE	/	/	+, Sp	/	Ro+	+	steroids, HCQ, MTX	India	Mediterr J Rheumatol. 2022;33(1):92‐93.
24	F	15	SLE	+	/	+, Sp	+	/	/	prednisone, MTX, belimumab	China	J Rheumatol. 2023;50(1):147.
25	F	51	SCLE	+	/	/	/	Ro+	/	prednisolone (40 mg/day)	USA	Cureus. 2023;15(5):e39631.

Abbreviations: AZA, azathioprine; CTX, cyclophosphamide; DLE, discoid LE; EM, erythema multiforme; F, female; HCQ, hydroxychloroquine; Hm, homogeneous; IVIG, intravenous immunoglobulin; /, Not stated; M, male; MI, mucosal involvement; MMF, mycophenolate mofetil; MTX, methotrexate; ND, not done; PL, perniotic lesions; RF, rheumatoid factor; SCLE, Subacute cutaneous LE; SLE, Systemic lupus erythematosus; Sp, speckled.

^a^ This reported case.

It has been reported that RS can be caused by pharmaceuticals (such as antituberculotics and antifungal medications), physical causes (such as UV light and radiation), infection, and so forth.^9^, ^13^ The patient in our case denied any prior drug usage and had no clear symptoms or indications of infection (particularly covid‐19 infection). In terms of treatment for RS, previous studies indicate that glucocorticoids, hydroxychloroquine, methotrexate, azathioprine, dapsone, cyclosporine, and so forth. are effective^13^, ^14^, ^15^, ^16^, ^17^ and that steroid decrease should be slower than in lupus. Researchers Shang et al.^18^ concluded that the level of some autoantibodies (anti‐dsDNA, anti‐Nucl, anti‐His, and anti‐C1q) might indicate how well SLE responds to therapy since they decrease following treatment. While anti‐Nucl is the most sensitive antibody for longitudinally evaluating SLE disease activity and therapy effectiveness, anti‐dsDNA performs best when assessing disease activity horizontally. According to Modi et al.^12^ RS was consistent with SLE or DLE with regard to the treatment strategy, dose–response relationship, and prognosis. There were findings that DLE in RS often manifested a chronic course, however the treatment could be more challenging if it coexisted with SLE. Fortunately, our case replied effectively to glucocorticoids and hydroxychloroquine. The patient's chilblain‐like skin lesions, EM on the hands and feet, and facial butterfly erythema all significantly improved after 2 weeks of treatment, along with the disappearance of proteinuria, normalization of liver enzymes, and recovery of leukopenia, anemia, and thrombocytopenia. Currently, this case is still being followed up in our hospital. In conclusion, we report an unusual case of RS in a 41‐year‐old female from China with EM lesions on limbs. This study may contribute to getting a better knowledge of Chinese RS patients for clinicians to recognize it, make an accurate diagnosis, and prescribe the appropriate course of treatment according to the LE procedure when LE patients develop EM‐like lesions.

## AUTHOR CONTRIBUTIONS

All the authors participated in the acquisition, analysis, interpretation of data or designed the study. All authors approved the final version to be published. *Writing original draft, conceptualization, and investigation*: Yanqiao Li. *Project administration and data curation*: Shi Cheng. *Methodology and validation*: Chuanpeng Ying and Ling Li. *Visualization, writing review and editing*: Guangxing Wang. *Supervision*: Xuejun Chen.

## CONFLICT OF INTEREST STATEMENT

The authors declare no conflict of interest.

## ETHICS STATEMENT

This study was approved by the research and ethics committee of the Sichuan Provincial People's Hospital. Informed consent was obtained from the patient involved in the study.

## Data Availability

All data are available from the corresponding author upon reasonable request.
